# Latest Humbug

**Published:** 1849-01

**Authors:** 


From the St. Louis Medical and Surgical Journal.

### LATEST HUMBUG.

*To the Editor of the Annalist:*

Dear Sir—I send you the statement which you asked of me, relating to a species of quackery, now, I regret to say, fast growing into favor with a portion of the public. I mean the treatment of diseases according to the assumed revelations of what is called *Mesmeric Clairvoyance.* I do so the more readily, that I believe if medical men would call the attention of their brethren, and through them of the public, to the disastrous consequences, both personal and pecuniary, of these monstrous impositions upon public credulity, to the duped, as seen by them in practice, their true character would be better appreciated; and if thereby the utter discomforture of the knaves who practice them were not to be effected, the number of victims would at least be greatly reduced. It surprises me very much, that the profession, which is constantly observing instances of the mischievous operation of popular delusions, and the injury done by them to the pockets and health of the poor gulls who suffer from them, should make no effort to expose their existence, and denounce their dangers. It is true that quackery can never be extinguished, and that, hydra-like, as fast as one head is stricken off, another of a new form rises in its place; but it is still something to remove, in some degree at least, a crying evil in one form, even though another replace it. The injury done to the particular race of defeated charlatans is not the less, because a new set, with some novelty, arise from their ruins.

If the public be warned of one danger, it is not the fault of the monitor that they incur another afterwards; and if, by the multiplication of instances, attention can be awakened towards any particular species of deception, the influence of the profession with the public is surely sufficient to lessen, at least, its sphere of operation. It is very right to caution the public to beware of a "mock auction," but it is the fault and folly of the green-horn, if, on being once warned, he walks off into another.

The case of which I spoke to you, was that of a very worthy and respectable man, of family and of general intelligence, who, having some slight rheumatic ailment, was persuaded by a friend to call upon a mesmeric practitioner. This person subjected him to the scrutiny of a *clairvoyante*, whom he had associated with him in practice, and who, under the influence of a magnetic sleep, asserted that the patient labored under a "complication of disorders." He was affected, according to her statements, with inflammation of the liver and kidneys, and a "*knotty condition of the nerves.*" ("His nerves were all drawn up into knots.") The treatment, which was based upon this erudite diagnosis, consisted in the administration, *ad libitum*, of a nostrum known by the name of "Sherwood's gold pills," varied occasionally by the exhibition of a Thomsonian purgative, the basis of which I believe to be mandrake, external frictions, the enveloping of the feet long and constantly in "skunk-cabbage" poultices, and long-continued and oft-repeated shocks by means of an electro-magnetic apparatus, which the patient purchased from his *medical* advisers, at a high price. The prostration and nervous irritability which ensued under this course of treatment, at length seriously alarmed both the patient, his family and friends; all of whom had been anxiously awaiting the promised amendment. I, who had been the former physician to the family, was then sent for, and the mesmerists dismissed from their attendance. Nor was it without a resort to conduct of the most unmistakable character, that they could be persuaded to forego the further plucking of the unfortunate pigeon whom they had ensnared, and reduced to a state of nervous exhaustion and debility, the most pitiable and alarming. I found my old friend in a most deplorable condition, cursing his own folly, and imploring me, with tears in his eyes, in a feeble and broken voice, to employ, if any hope yet remained, my best skill, to save him from the consequences of his delusion, and the fearful experiment which he so much regretted to have taken. It gives me pleasure to add, that in time he recovered, a wiser man; nor is it likely that he, or his, will ever, hereafter, give ear to any of the mummeries and absurdities which defile the fair temple of our Science.

You are at liberty to use the facts here stated in any way that you see fit; and if the narration contribute in the least to the overthrow of this and every other class of charlatans, who, to the disgrace of our age and country, prey with impunity upon the community, to the injury of its health, its morals, and its happiness, not less than to that of legitimate medicine, no one will rejoice more sincerely than

Yours, very truly, G.

P. S. I need not say that my unfortunate patient paid dearly, in more senses than one, for his experience. Every "session" of the clairvoyante incurred the moderate charge of $10,00. No wonder quackery flourishes when men of sense can be found to pay for it so liberally.

